# 3D bioprinting of multi-layered segments of a vessel-like structure with ECM and novel derived bioink

**DOI:** 10.3389/fbioe.2022.918690

**Published:** 2022-08-19

**Authors:** Federica Potere, Beatrice Belgio, Giorgio Alberto Croci, Silvia Tabano, Paola Petrini, Gabriele Dubini, Federica Boschetti, Sara Mantero

**Affiliations:** ^1^ Laboratory of Biological Structure Mechanics (LaBS), Politecnico di Milano, Department of Chemistry, Materials and Chemical Engineering “Giulio Natta”, Milan, Italy; ^2^ Division of Pathology, Fondazione IRCCS Ca’ Granda Ospedale Maggiore Policlinico, Milan, Italy; ^3^ Department of Pathophysiology and Transplantation, Università, Degli Studi di Milano, Milan, Italy; ^4^ Laboratory of Medical Genetics, Fondazione IRCCS Ca’ Granda Ospedale Maggiore Policlinico, Milan, Italy; ^5^ Politecnico di Milano, Department of Chemistry, Materials and Chemical Engineering “Giulio Natta”, Milan, Italy

**Keywords:** tissue engineering, tubular constructs, decellularisation, bioink, 3D bioprinting

## Abstract

3D-Bioprinting leads to the realization of tridimensional customized constructs to reproduce the biological structural complexity. The new technological challenge focuses on obtaining a 3D structure with several distinct layers to replicate the hierarchical organization of natural tissues. This work aims to reproduce large blood vessel substitutes compliant with the original tissue, combining the advantages of the 3D bioprinting, decellularization, and accounting for the presence of different cells. The decellularization process was performed on porcine aortas. Various decellularization protocols were tested and evaluated through DNA extraction, quantification, and amplification by PCR to define the adequate one. The decellularized extracellular matrix (dECM), lyophilized and solubilized, was combined with gelatin, alginate, and cells to obtain a novel bioink. Several solutions were tested, tuning the percentage of the components to obtain the adequate structural properties. The geometrical model of the large blood vessel constructs was designed with SolidWorks, and the construct slicing was done using the HeartWare software, which allowed generating the G-Code. The final constructs were 3D bioprinted with the Inkredible + using dual print heads. The composition of the bioink was tuned so that it could withstand the printing of a segment of a tubular construct up to 10 mm and reproduce the multicellular complexity. Among the several compositions tested, the suspension resulting from 8% w/v gelatin, 7% w/v alginate, and 3% w/v dECM, and cells successfully produced the designed structures. With this bioink, it was possible to print structures made up of 20 layers. The dimensions of the printed structures were consistent with the designed ones. We were able to avoid the double bioink overlap in the thickness, despite the increase in the number of layers during the printing process. The optimization of the parameters allowed the production of structures with a height of 20 layers corresponding to 9 mm. Theoretical and real structures were very close. The differences were 14% in height, 20% internal diameter, and 9% thickness. By tailoring the printing parameters and the amount of dECM, adequate mechanical properties could be met. In this study, we developed an innovative printable bioink able to finely reproduce the native complex structure of the large blood vessel.

## 1 Introduction

Large diameter arterial blood vessels are characterized by an average inner diameter of 3 cm and an average length of 35 cm. The arterial wall comprises three concentric layers: tunica intima, tunica media, and tunica adventitia. Each layer has specific protein and cell composition and performs specific functions. The tunica intima, the innermost layer, consists of a monolayer of endothelial cells anchored to a basement membrane, beneath which lies the elastic lamina. This layer helps maintaining the vascular tone and forms a tight barrier between the vessel lumen and the wall thus preventing clot formation, infection, and inflammation. Tunica media, the medial layer of the artery, is mainly composed of smooth muscle cells, elastin and collagen fibers. The collagen and elastin matrix coupled with contraction and dilation of muscle cells provide mechanical strength to the intimal layer. The outermost layer of the vessel wall is the adventitia, a connective tissue made of a loose matrix of collagen and elastin with embedded fibroblasts (Goins et al., 2019). Adventitia acts as a connecting substrate between the blood vessel and the surrounding tissue. In addition, it plays a structural role.

Several vascular diseases such as peripheral arterial disease, abdominal aortic aneurysm, and aortic dissection may affect blood vessels and impair blood flow (McAllister, 1989). These pathological conditions likely culminate in life-threatening events, such as bleeding, stroke, and infarction. The implantation of vascular grafts is the current treatment strategy in clinical practice to replace damaged vessels and revascularize. Autogenous vein and arterial grafts, such as great saphenous veins and internal mammary and radial arteries, remain the gold standard conduits for vascular reconstruction. However, the complex invasive surgical procedure, the extent of the lesion size and their limited availability hinder the use of autologous grafts. For these reasons, a new approach has emerged: the development of synthetic vascular prostheses. Polyethylene terephthalate is the most used synthetic material for the fabrication of large diameter vascular grafts (Mehta et al., 2011) However, its usage is associated with the long-term administration of anticoagulant drugs that may have high risks of bleeding and hemorrhage. In addition, late complications may arise from postoperative infections, material degradation, and loss of the structural integrity of the graft (Mehta et al., 2011). Therefore, new options for more efficient replacement of blood vessels are needed. Nowadays, it is well known that cells play a key role in the formation and maintenance of functional blood vessels (Devillard and Marquette, 2021). Hence, research has focused on tissue-engineered vascular conduits.

To date, many biofabrication techniques, including electrospinning, decellularization of xenogeneic vessels, 3D printing, and melt electrowriting have been explored (Xue et al., 2019) (Rabionet et al., 2018) (Yuan et al., 2012) (Dalton, 2017) (Tresoldi et al., 2019) (Devillard and Marquette, 2021). However, achieving a homogenous cell distribution within the vascular layers remains challenging (Devillard and Marquette, 2021). Indeed, none of the previously mentioned methods allows to selectively and precisely control the location of cells in a 3D in vivo-like architecture. Moreover, the production of multilayered functional vessels with these techniques implies a multi-step complex process, during which every layer needs a specific maturation time. To overcome these main drawbacks, 3D bioprinting technique have been recently exploited, as it provides incomparable advantages (Gao et al., 2019). This new technology enables to obtain of 3D cellularized constructs with distinct layers in a single fabrication step, thus replicating the hierarchical complex structure of vascular tissue (Moghaddam et al., 2021) (Schwab et al., 2020). Among the many bioprinting strategies, pneumatic extrusion-based bioprinting seems the most promising to produce large vessels characterized by cell layers of centimeter-size tubular architectures. During this process, the bioink, a formulation of cells suspended in a biomaterial, is extruded through a nozzle through a pneumatic system and deposited layer-by-layer onto a receiving substrate. A wide choice of bioinks can be employed with this technology. The most used types of bioinks are based on hydrogels, polymeric structures swollen in water, whose composition can be designed and tailored to reproduce the extracellular environment of biological tissues. However, the weak mechanical properties of hydrogels limited their applicability for vascular tissue engineering. A possible solution could be represented using a synthetic material with the function of a supportive scaffold (Jorgensen et al., 2020) (Bishop et al., 2017). For instance, Jang and his group produced a three-layered vascular scaffold using polycaprolactone (PCL) as support (Jang et al., 2020). In particular, a layer of bioink, composed of cells and 3% sodium alginate, was printed between two layers of PCL to guarantee an adequate support. Similarly, Jia et al. used poly (- ethylene glycol)-tetra-acrylate (PEGTA) as a supportive material for a cell-responsive bioink, formed by gelatin methacryloyl (GelMA) and sodium alginate. However, the bioinks used in these studies are not representative of the native extracellular matrix (ECM) components. The principal components of the ECM such as collagen, elastin, microfibrils, proteoglycans, glycosaminoglycans (GAGs), and various growth factors are essential for preserving large vessel structure in vascular tissue-engineered (Carrabba and Madeddu, 2018) (Mantero et al., 2007). Recently, bioinks based on decellularized extracellular matrix (dECM) have been developed. In fact, decellularization process allows preserving the native vessel microenvironment, created by the ECM, which promotes cell growth and a non-immunogenic tissue while removing cellular and nuclear components - with a particular interest to DNA and RNA - from tissue (Kabirian and Mozafari, 2020) (Edgar et al., 2020) (Jang et al., 2016) (Pellegata et al., 2012). Therefore, this strategy allows to obtain a bioink composed of biochemical cues present in the native environment.

This work presents the development of a novel bioink composed of dECM and natural hydrogels able to reproduce large vessel substitutes compliant with shape, functionality, and integrity requirements of the native tissues. Our strategy combines the advantages of the 3D bioprinting and decellularization process. First, we optimized the decellularization protocol. The obtained dECM was then incorporated in a bioink whose formulation guaranteed its printability thus facing one of the main challenges with extrsuion-based 3D-bioprinting (Paxton et al., 2017) (Schwab et al., 2020) (Sardelli et al., 2021). Finally, we verified the bioink biocompatibility and cell infiltration monitoring cell growth in 3D printed constructs over time.

## 2 Materials and methods

First, we have optimized a protocol to decellularize the native porcine aorta.

The treated samples were tested to evaluate the efficiency of the decellularization protocol, and subsequently, powdered by cryo-milling under N_2_. Then, dECM powders were solubilized by pepsin digestion. To develop the bioink, several solutions, based on different concentrations of gelatin, alginate, and digested dECM, were studied. After designing the geometrical model of the aorta structures by SolidWorks, the constructs slicing was performed using the software CELLINK HeartWare, which allows generating the G-Code. Finally, the constructs were 3D bioprinted using the Inkredible + (Cellink, A Bico Company, Sweden), and the three layers were identified with different colored bioinks to highlight functional areas in accordance with the native anatomy.

### 2.1 Native aortic segment decellularization

**FIGURE 1:**
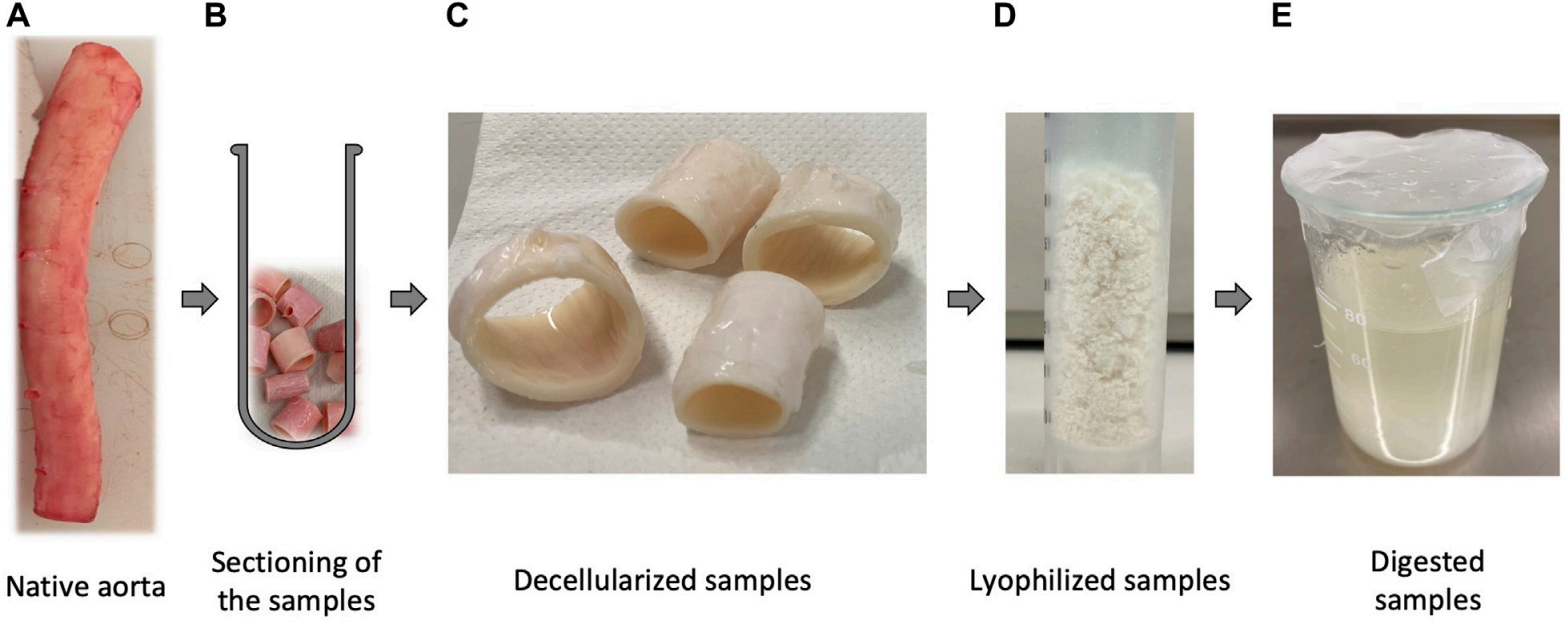
Chronological pathway from the native tissue to the solution of the digested porcine native aorta. In particular: **(A)** Native porcine aorta provided by a local abattoir; **(B)** Samples of native aorta cut into segments of approximately 2 cm, ready to be treated with the decellularization process; **(C)** samples after undergoing the decellularization protocol; **(D)** powder of the treated samples produced by cryomilling under N_2_; **(E)** solution of the decellularized powder performed through enzymatic digestion.

**FIGURE 2:**
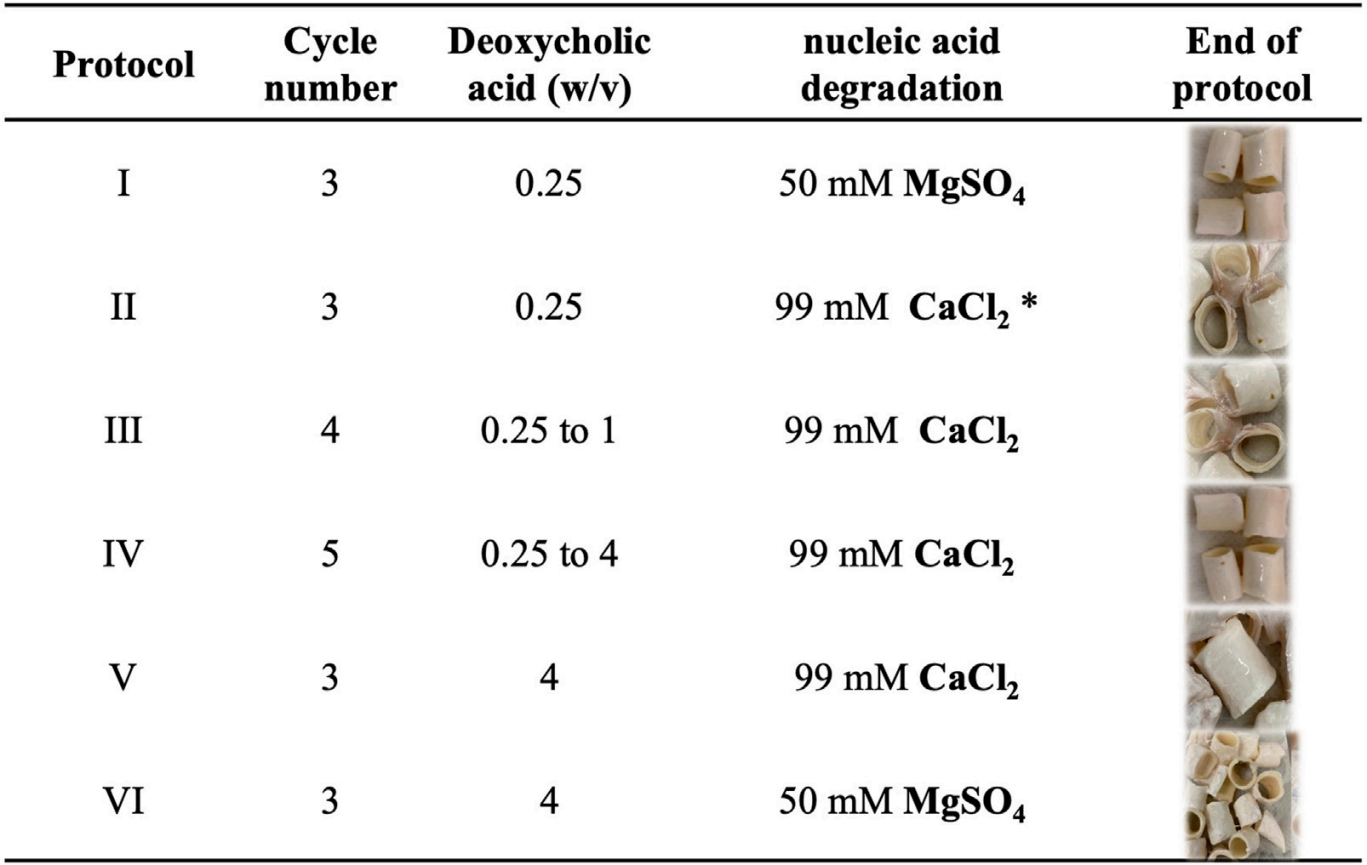
Optimization of the decellularization protocol. In particular (column 2) the number of cycles performed and the percentage of deoxycholic acid dissolved in dH_2_O for each protocol tested, (column 3) the degradation step of nucleic acids made with 22.5 mg/l DNAse. Protocol I and VI provide the use of MgSO4 as salt, unlike the other protocols that use CaCl_2_. Protocol two provides for the addition of 0.9 NaCl. In column 5, the macroscopic appearance of the samples at the end of the protocol.

All the experiments were performed on aortas obtained from 6-months female and male pigs kindly provided by the local abattoir, previously instructed on harvesting and preserving tissues. Based on the literature review, the path of the decellularization process consisted of cutting the native porcine aorta (Figure 1A) into segments of about 2 cm in length (Figure 1B) and washing them in deionized water containing 1% penicillin/streptomycin at 4^∘^C to cause cell lysis by osmotic shock. Subsequently, the first and second steps of the decellularization were performed using deoxycholic acid at the different concentrations reported *in* Figure 2 to solubilize cellular and nuclear membranes, DNAse (22.5 mg/l) to catalyze the hydrolysis of DNA and RNA, combined with MgSO_4_ (50 mM), or CaCl_2_ (99 mM), according to the six detergent-enzymatic protocols (Figure 2). In addition, after each treatment, the samples were rinsed three times in PBS (Gibco, United Kingdom) containing 1% penicillin/streptomycin (all reagents Sigma-Aldrich, St. Louis, MO, United States). The number of cycles varied between one and five for each protocol (Figure 2) (Batioglu-Karaaltin et al., 2019) (Zambaiti et al., 2019) (Pellegata et al., 2015). The decellularized samples are reported in Figure 1C.

#### 2.1.1 Histology

Histologic examination was performed on specimens fixed in 10% buffered formalin for 24 h and paraffin embedded. For each specimen, 4 µm-thick slides were stained with haematoxylin-eosin and assessed for the presence of residual vital cellularity, residual nuclei, and preservation of the tissue architecture.

#### 2.1.2 DNA Quantification

DNA was extracted from frozen tissues using the Gentra Puregene Tissue Kit (Qiagen), following the manufacturer’s instructions. Briefly, about 5–10 mg of frozen tissue were placed in a tube containing the cell lysis solution and homogenized using the Tungsten Carbide Beads (Qiagen) by a TissueLyser (Qiagen). Cell lysis was carried out at 65°C for 20 min. After complete cell lysis, Puregene Proteinase K was added to samples at 56°C for 1 h. Proteins were precipitated by adding Protein Precipitation Solution to eliminate them. Finally, DNA was recovered and precipitated with isopropanol, washed by 70% ethanol, and resuspended in 100 ul using the DNA hydration solution. DNA quantification was performed with QIAxpert (Qiagen) and Nanodrop (Thermo Fisher Scientific) systems. When spectrophotometers failed to detect DNA in a sample, to reveal even minimal DNA traces, a PCR was performed, using primers amplifying DNA fragments of 100, 200, 300, and 400 bps (primer sequences available on request). A sample was defined as “negative” (= absence of DNA) when no PCR products were obtained on agarose gel.

### 2.2 ECM gel bioink design

The biomaterials of the bioink used in this work were gelatin, alginate, and vascular dECM, mixed at different ratios. To obtain the vascular dECM, the decellularized vessel had to be lyophilized and then digested. After the decellularization protocols, the treated samples (Figure 1C) were cryomilled under N_2_ to produce a powder (Figure 1D). Subsequently, the powder was digested with pepsin (Figure 1E) following this protocol: the powdered dECM and the pepsin were dissolved in HCl (0.01 M) according to the ratio of 1:0.1, for 96 h. In the end, to stop the digestion of pepsin and restore the pH to the physiological value of 7.4, drops of 10 M NaOH were added. The solution was stored at 4 °C to avoid gelation which occurs at 37°C (Freytes et al., 2008) (Park et al., 2018).

To increase the printability and shape fidelity of the bioink, the digested powder was mixed with alginate and gelatin. First, the proper concentration of alginate and gelatin was evaluated. In brief, the bioink was obtained by mixing different percentages, ranging from 4% to 10% for both gelatin and alginate, as follows: gelatin was dissolved in distilled water under continuous stirring at 60°C for about 15 min and then alginate was added, keeping stirring constant for 1 hour at 37°C (Di Giuseppe et al., 2018). The powders of gelatin and alginate were disinfected in 100% ethanol for 8 h. Finally, dECM solution was mixed with the suspension of alginate and gelatin (0%, 3%, 5%, and 10% w/v). For printability assessment, 0.02 g of powder dye (blue and red) were added to the bioink to have the hierarchical 3D printed structure distinguishable.

### 2.3 Cell culture

Mouse fibroblast cells (L929; Thermo Fisher Scientific, United Kingdom) were expanded (passage 12–13) and used in this study. L929 were cultured in Dulbecco’s Modified Eagle Medium (DMEM, Gibco™, United Kingdom) supplemented with 10% (v/v) fetal bovine serum (FBS) and 1% (v/v) penicillin-streptomycin solution (Sigma-Aldrich, United States). The cells were incubated at 37°C, 5% CO2, and 95% relative humidity.

### 2.4 3D bioprinting of multi-layered vessel

The constructs were bioprinted using the Inkredible + (Cellink, A Bico Company, Sweden). It is a pneumatic-based extrusion system with dual print heads and built-in UV LED curing with a three-axis stage system. For bioprinting the constructs, we utilized two cartridges. In this work both the cartridges were loaded with the bioink consisting of 8% (w/v) gelatin, 7% (w/v) alginate, 3% (w/v) dECM. Nozzles with 400 μm were used for laden bioink. Prior to bioprinting, a customized printing code was developed to print the hydrogels in the form of a tubular construct with the L929-laden bioink. Cells at passage 13 were encapsulated in the bioink with a cell density of 2 × 10^6^ cells ml^−1^ to bioprint all the three layers of the construct. The dispensing pressure was 100 kPa and the velocity was 20 mm min^−1^. Moreover, the percentage of the desired infill was set at 50% for the first layer and then at 0% for the entire structure due to the tendency of bioink to collapse. The printed construct was cross-linked with 1% (w/v) CaCl_2_ for 10 min at the end of the printing process. After that, the structure was cultured in DMEM. Differently colored bioinks were used: the blue/red bioinks were used to visualize and evaluate the printability of two different layers, blue for both the intima and adventitia layers, while the red one for the media layer.

### 2.5 Cell viability assay

Cell viability in the 3D bioprinted constructs (*n* = 8) was determined using a Live/dead viability/Cytotoxicity kit (Invitrogen, United States) according to the manufacturer’s instructions. Briefly, the samples were collected and washed with PBS and then incubated in staining solution containing 0.5 μl ml−1 calcein-AM and 2 μl ml−1 of ethidium homodimer at 37°C for 45 min. Fluorescence microscopy Eclipse Ti2-E (Nikon, Japan) was used to evaluate the live (green) and dead (red) staining cells in the printed constructs. Three random views from each sample were photographed and analyzed using Fiji ImageJ software.

### 2.6 Rheological tests

The viscoelastic properties of hydrogels were characterized by a modular rheometer (MCR 502e, Anton-Paar, AT). If not differently stated, all tests were performed adopting a double plate geometry with 25 mm diameter plates (Anton-Paar, serial number: 52,890). Briefly, the sample was loaded onto the rheometer stage and the 25-mm steel plate geometry was lowered until contact with the surface of the sample. The storage modulus G’ and loss modulus G” were measured for each test using a frequency sweep test. The frequency sweep was performed at a strain amplitude of 1%, at a logarithmic increasing of frequency set between 0.05 (*ω* = 0.314 rad/s) and 20 Hz (*ω* = 125.7 rad/s). The rheology of the bioink was tested according to the following variables: crosslinking, and temperature.

### 2.7 A novel geometrical model of the 3D Bioprinted large blood vessel

**FIGURE 3:**
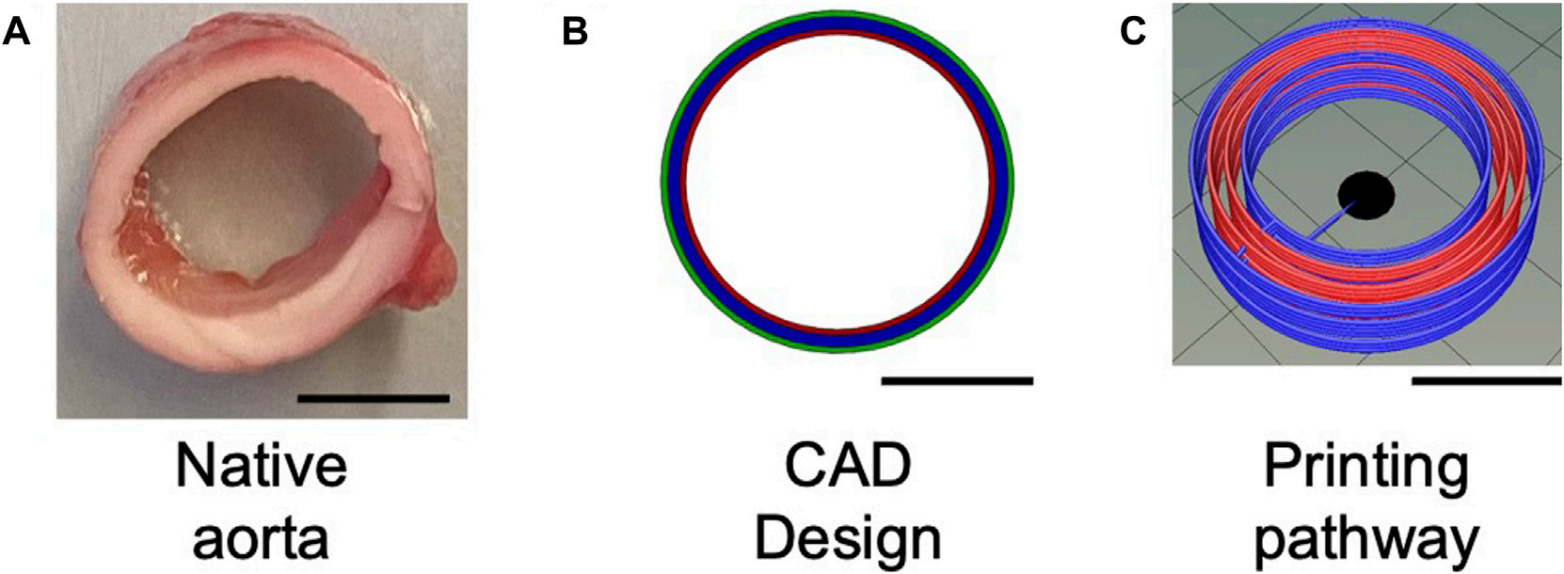
A geometrical model of the hierarchical structure of the aorta. In particular: **(A)** Native aorta; **(B)** CAD model of the aorta construct, based on the real anatomical dimensions of the aorta; **(C)** Printing pathway. In **(B)** the three layers of the native aorta are identified: in red the intima layer, in blue the media layer, and in green the adventitia layer, whereas in **(C)** the latter is in blue as well. (Scale bar = 10 mm).

The pre-processing phase began with the design of the CAD model of the aorta structure. The design of the construct was performed with the CAD software SolidWorks (Dassault Systèmes SOLIDWORKS Corp., Waltham, MA, United States). It allowed the realization of a 3D structure through a parametric and customizable system. The aorta models were designed considering the anatomical dimensions (diameter is generally 20–30 mm, with a thickness of about 2 mm). The final design of 3D constructs is shown in Figure 3. To reproduce the hierarchical structure of the aorta (Figure 3A), each component was then designed individually: one layer each for the adventitia (green in Figure 3B) and intima (red in Figure 3B), and two layers for the media (blue in Figure 3B). In the native aorta, the media layer is thicker compared to the other two layers. The next step regarded the slicing of the construct performed by the CELLINK HeartWare software (Figure 3C). The program receives files in STL format in input and returns the files in G-code format, after processing and stratification of the construct according to the set parameters. The latter is a code that provides the coordinates to drive the extruders during the printing of the 3D constructs. In this phase, it is possible to set all the parameters properly.

The parameter optimization led to setting the nozzle diameter at 22 G, the height of each layer at 0.41 mm.

## 3 Results

### 3.1 Native aortic segments decellularization

**FIGURE 4:**
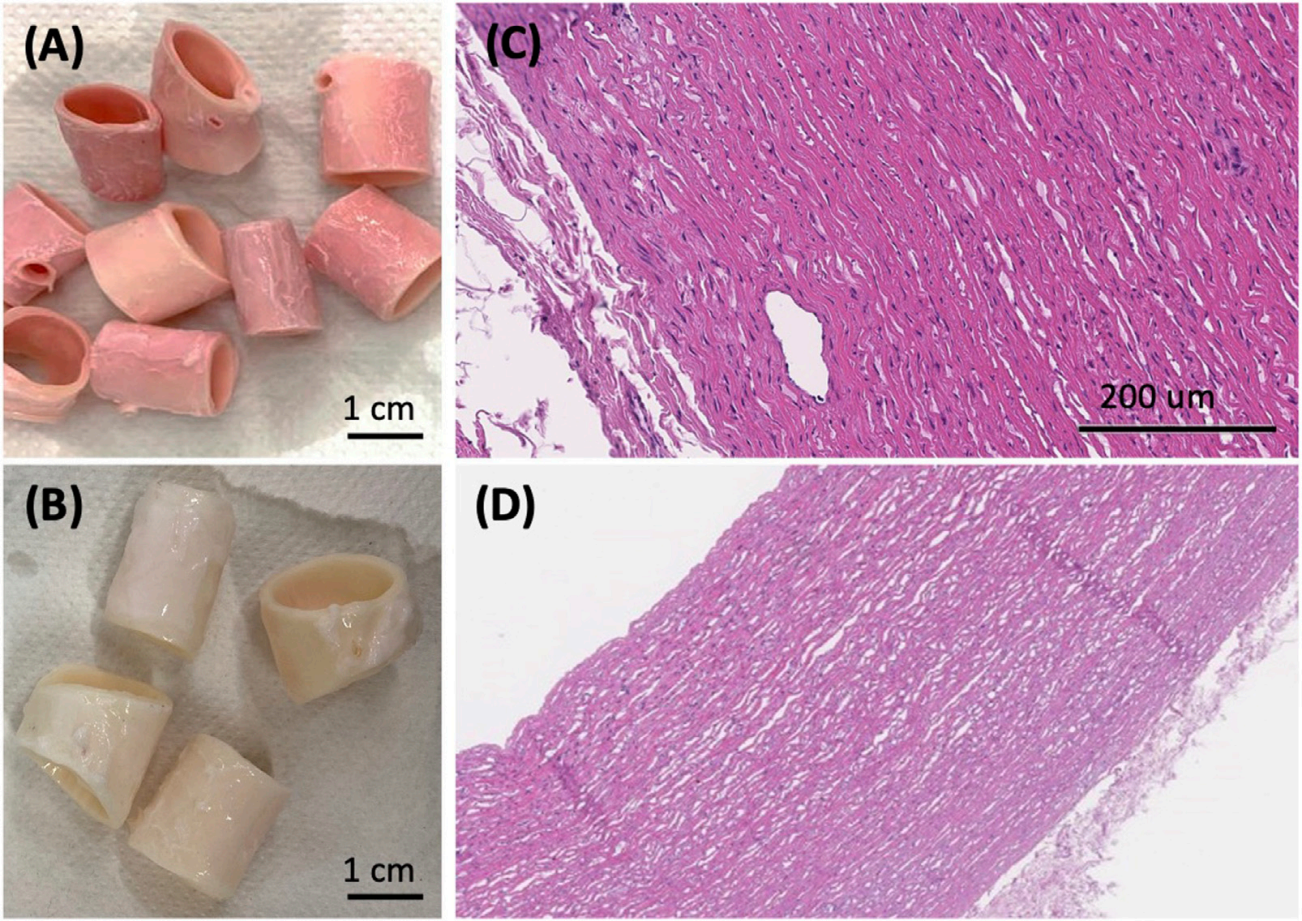
**(A)** Macroscopic appearance of the native aorta and **(B)** the decellularized samples showing the preservation of the macroscopic structural stability after the decellularization process. Haematoxylin and eosin staining shows the native arterial wall [**(C)**, H/E, 40x], and the decellularized arterial wall [**(D)**. H/E, 200x], where no nucleated elements are detected after 3 decellularization cycles.

The decellularization process aims at removing the tissues cellular component while preserving the tissue native structure and integrity. However, the process is complex, especially when it is applied to an entire organ. Several decellularization protocols were tested with the purpose of achieving adequate decellularization according to the evaluation parameters to be met. In particular, the quantity of DNA must be less than 50 ng/mg of tissue and the DNA fragment length less than 200 bp (base pairs). Moreover, the DNA and RNA components must be not visible in hematoxylin and eosin staining (Gilpin and Yang, 2017). After the sectioning of the sample, we varied the percentage of deoxycholic acid (Figure 2) from 0.25% to 4% to define the suitable amount to solubilize cellular and nuclear membranes. Subsequently, in the degradation phase of nucleic acids, the amount of DNase was kept constant among the various protocols, but the dissolved salt was varied. Specifically, in protocols I and IV MgSO_4_ (50 mM) was used; instead in the other protocols CaCl_2_ (99 mM) was used. The optimization of the process took place with the extraction and quantification of DNA until the value was reached. Protocol II showed the best results in terms of nucleic acid removal. Therefore, it was chosen as an optimized decellularization protocol. Firstly, the samples were washed for 1 h in deionized water. The following steps were 4 h with 4% w/v deoxycholic acid, 1 h in deionized water, 3 h 22.5 mg/l DNase in 0.9% w/v sodium chloride, and 1.11 g/l calcium chloride. After the decellularization process, all the samples were preserved at 4°C, in PBS with 1% penicillin/streptomycin. Results indicate the reduction in the DNA content indicating the cell removal. In particular, the quantity of DNA resulted in less than 50 ng/mg of tissue and the DNA fragment length less than 200 bp (base pairs). Moreover, reduction in nucleic acids components was corroborated at histology in the form of a reduced number of nuclei and, notably, of vital cells, particularly in the intimal and adventitial layers of the vessel (Figures 4C,D). On the contrary, no remarkable structural changes were observed in the extracellular matrix, as a well-preserved layering, without fragmentation of elastic fibers, could be appreciated. From the macroscopic appearance of the tissue, it is worth noting that the resulting structure was preserved without any evident failure even after three decellularization cycles (Figure 4B), comparing it with the native tissue (Figure 4A). Immediately at the end of the cycles, a first qualitative assessment of the color of the samples showed the white appearance (Figure 4B) of the decellularized sample, different from the pink macroscopic appearance of the native tissue (Figure 4A).

### 3.2 3D bioprinting process of a novel large blood vessel segments

**FIGURE 5:**
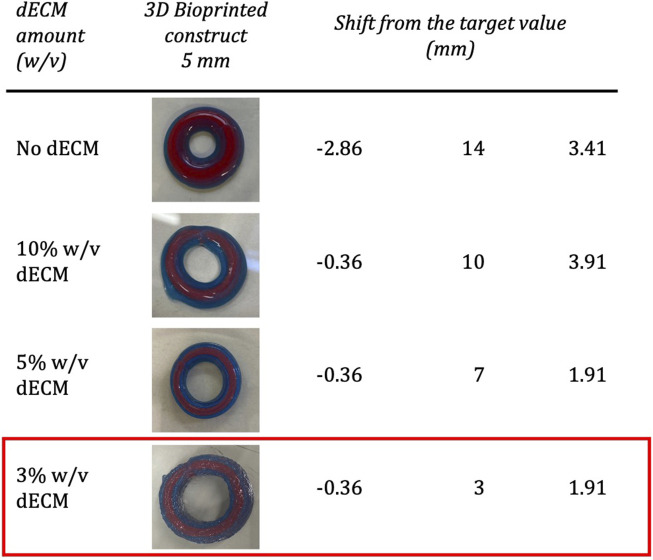
Study of the dimensions and shape fidelity of the bioink, reporting (column 1) the amount of dECM added to the base bioink composed of 8% w/v gelatin, 7% w/v alginate; the images of the 3D bioprinted constructs (column 2), and the shift from the target value, obtained by comparing the thickness (T), the inner diameter (D_i_) and the height of the designed construct with the printed construct. Specifically, the designed dimensions are Thickness = 4.64 mm, D_i_ = 20 mm, Height = 4.91 mm. The ECM bioink composed of gelatin 8% (w/v), alginate 7% (w/v) and dECM 3% (w/v) was selected.

Qualitative assessments of the shape fidelity were carried out for each bioink composition. The analysis of the filament diameter, the pore index, and the filament collapse was performed by first extruding the bioink through a syringe and then printing test grids and coils. From this first assessment, the association of 8% w/v gelatin, and 7% w/v alginate resulted in bioprinted structures with high fidelity to the imposed geometry and low post-printing spreading. As shown in Figure 5, the increasing amount of dECM modified, even at the macroscopic level, the printed construct.

**FIGURE 6:**
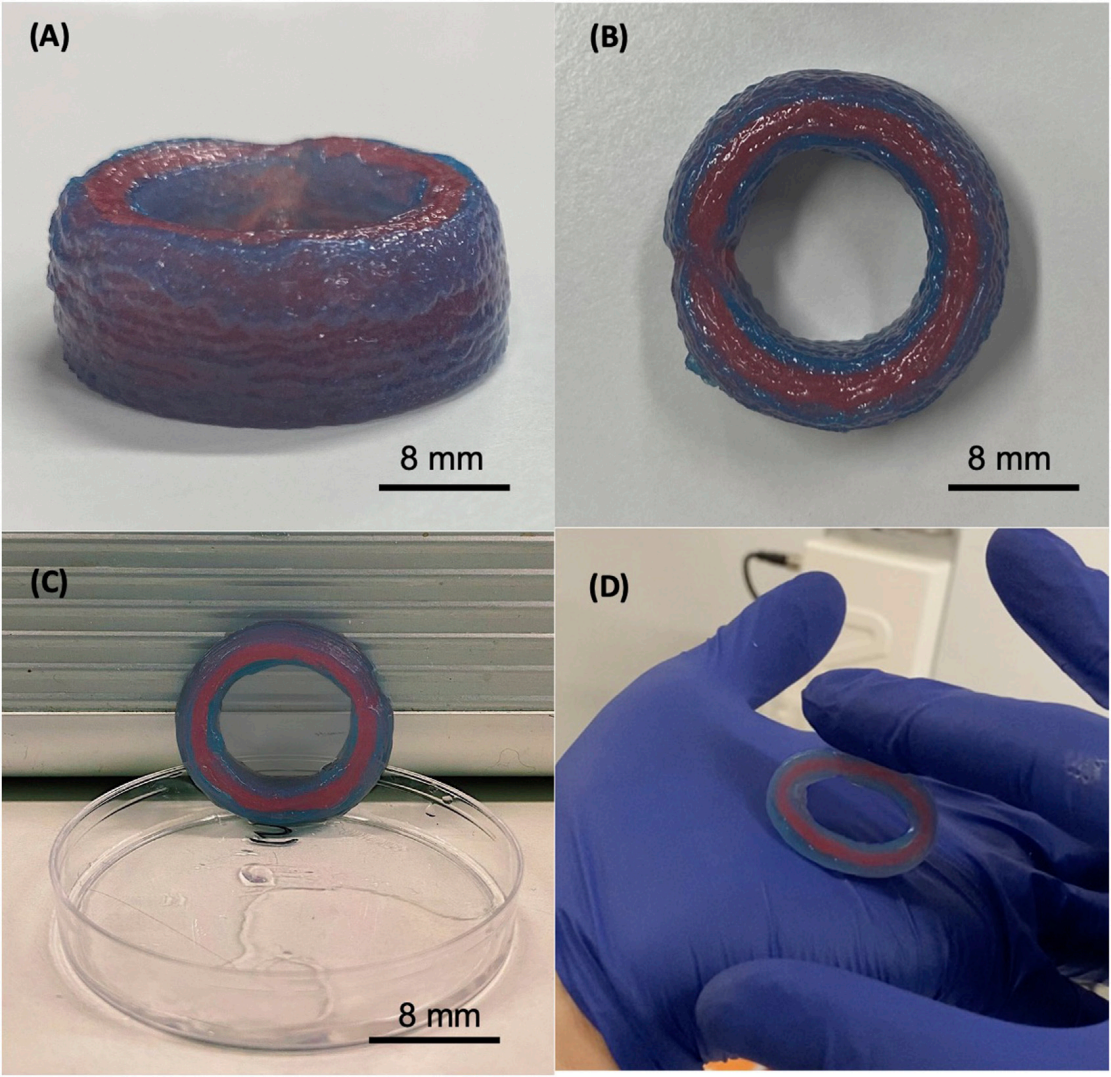
The 3D bioprinted construct composed by 8% w/v gelatin, 7% w/v alginate, and 3% w/v dECM. Printing settings: pressure = 100 kPa and velocity 20 mm/s. The height of the produced sample is 9 mm **(A)**, the internal diameter is 17 mm and its thickness is 4 mm **(B)**. The construct maintains the shape and does not collapse under its weight **(C)**, and it results elastic and stable in the handling and under cycle stress applied manually **(D)**.

According to the evaluated parameters, the ECM bioink composed of 3% w/v dECM was selected for printing a segment of the tubular construct of approximately 10 mm in height. Considering the geometrical structures, the dimensions of the printed structures were very close to those established in the design phase (Table 1). In addition, by combining the described bioink with a post-printing crosslinking with 1% CaCl_2_, it was possible to 3D bioprint the desired structures up to 20 layers, corresponding to approximately 10 mm in height (Figures 6A,B). It is important to emphasize how the printed structure successfully maintained its shape without breaking, despite the externally imposed deformation, returning to its initial structure (Figures 6C,D).

**TABLE 1:** Comparison between the modeled (geometrical model) and the 3D bioprinted construct main geometrical dimensions.

Aorta	Thickness (mm)	Internal diameter (mm)	Height (mm)
Modeled construct	4.64	20	9.91
3D Bioprinted construct	4.00	16.00	9.00

Another challenge was to reproduce the multilayered structure. The bioink composition and the bioprinting process were set according to this aim. We were able to bioprint the two distinct layers, as demonstrated by employing two different cartridges, containing bioinks colored in blue and red. This is proof of the concept of the possibility to print two different cell types loaded in different cartridges and to confine, in the first phases of the scaffold colonization, two different cell layers, without inducing mixing between layers in the bioprinting phase.

### 3.3 Cell viability assay

**FIGURE 7:**
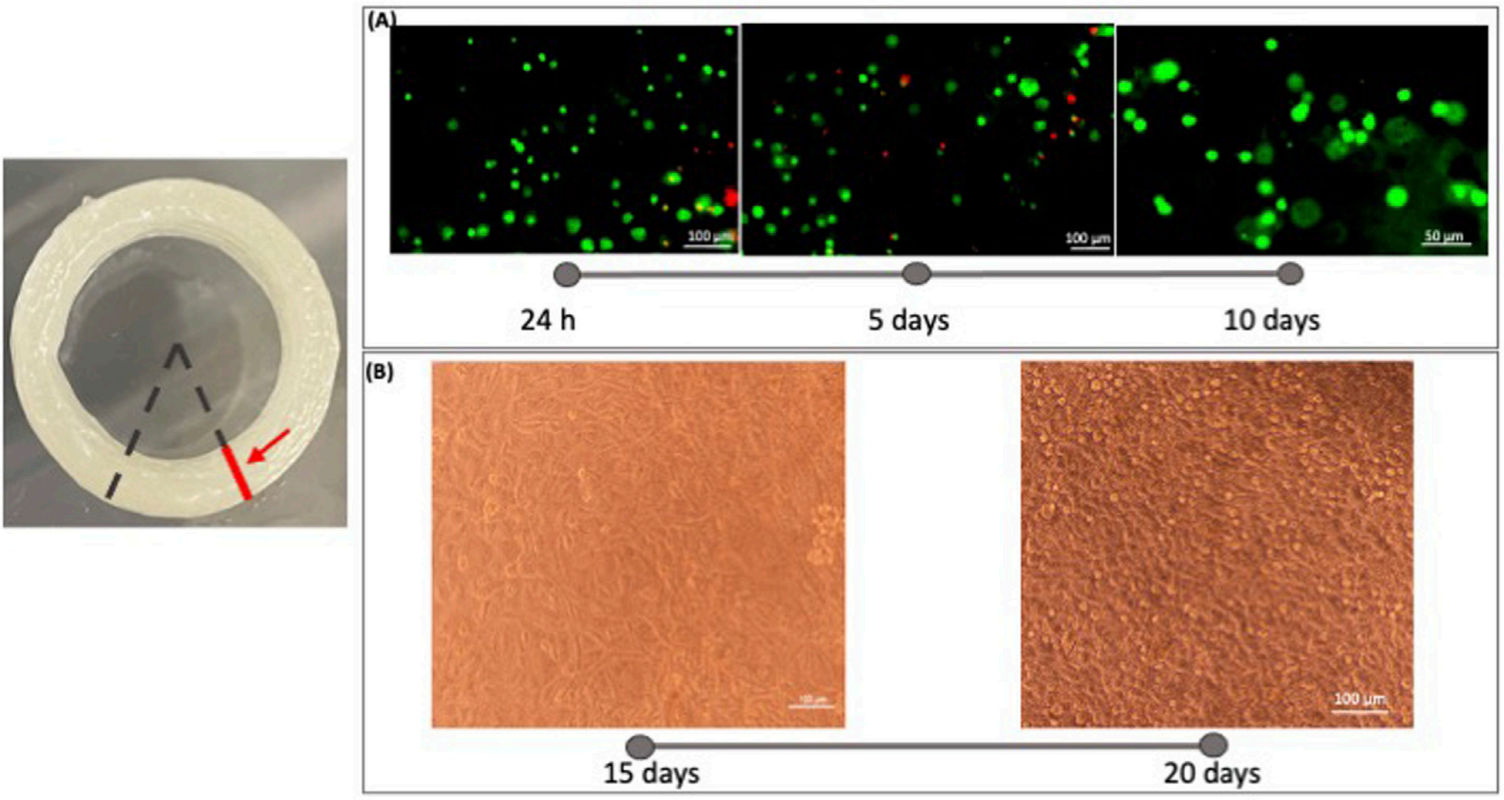
Microscope images were acquired on the sample section. The red line indicates the section plane on which the acquisition took place. The analysis of cell viability in the 3D bioprinted constructs was performed using a Live/Dead viability/Cytotoxicity kit (Invitrogen, United States). **(A)** Fluorescence images were taken at 1 day, 5 days, and 10 days to evaluate the live (green) and dead (red) staining cells in the printed constructs with microscopy Eclipse Ti2-E (Nikon). Three random views from each sample were photographed and analyzed using Fiji ImageJ software. From these images, we can say that the developed bioink is biocompatible and cytocompatible. **(B)** Images of the cell infiltration were captured at 15 and 20 days of culture. Comparing the images of different time points we can appreciate a cell growth into the 3D Bioprinted construct.

The Live&Dead assay was applied to estimate the cell viability in bioprinted blood constructs. The live and dead results were examined by fluorescence microscopy after live/dead staining (Figure 7A) shows the fluorescence images of day 1, day 5 and day 10 at ×10 magnifications. Cells retained their printed position at day 1 after printing. After 5 days in culture, the cells started to extend and connect with each other. From these images, we can say that the developed bioink is biocompatible and cytocompatible. From figure B, after 20 days of culture, more cells extended than that on day 15. These results demonstrate that high cell viability can be achieved in the printed tubular constructs after printing (Figure 7B). Comparing the images of different time points we can appreciate a cell growth into the 3D Bioprinted construct.

### 3.4 Rheological characterization

**FIGURE 8:**
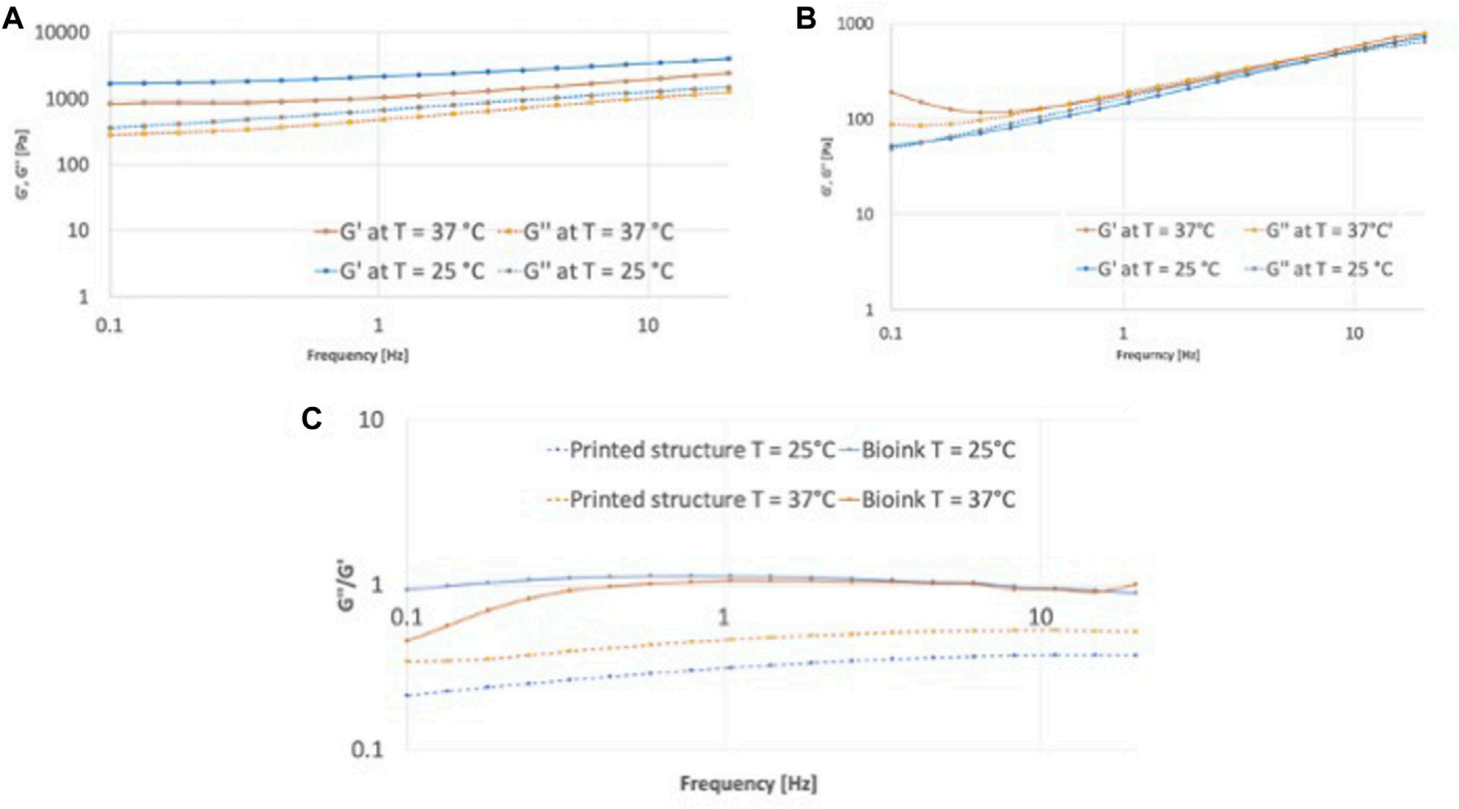
Rheological characterization in frequency sweep mood of the bionk and the printed structure. Comparison of storage modulus G′ and loss modulus G″ at T = 25°C and T = 37°C of the printed structure in **(A)** and of the bioink in **(B)**; **(C)** tanδ, defined as the ratio of G”/G′ of bioink and printed structure at T = 25°C and T = 37°C.

Rheological characterization of hydrogels allowed to characterize the flow characteristics of the bioinks and the bioprinted crosslinked hydrogel. The storage modulus (G′) and loss modulus (G″) of the bioink were typical of a liquid with overlapping values regardless the temperature (Figure 8A). The crosslinked hydrogel was less dependent on frequency, with a predominant solid-like behavior, as indicated by the storage modulus higher than the loss modulus and tan *δ* < 1 (Figure 8C) at all the considered frequencies. The increase of test temperature decreased the values, possibly due to the gelatin phase transition, although retaining the solid-like behavior with tan *δ* < 1.

## 4 Discussion

3D bioprinting has been investigated over the last years to obtain a 3D customized structure with several distinct layers to replicate the hierarchical structure of tissues (Habib and Khoda, 2018) (Ribeiro et al., 2018). Decellularization has also been studied as a method to remove cells from tissue structures, preserving the mechanical and biological properties of ECM. Indeed, the engineered ECM should be able to mimic the mechanical, biological, and chemical characteristics of the native ECM properly (Kim et al., 2020). However, the extraction process and the creation of a decellularized scaffold are complex and must be tailored to the specific tissue and monitored to prevent excessive damage and loss of integrity (Heid and Boccaccini, 2020) (de Melo et al., 2020) (Marques et al., 2019). Previously the decellularization strategies aimed at decellularizing entire organs suitable for recellularization to obtain scaffolds with the original native structure. Recently, studies have been focused on the creation of gel forms of decellularized tissues (Rajab et al., 2020) (Gilpin and Yang, 2017) (Edgar et al., 2020) (Moghaddam et al., 2021).

This study presents a multilayered tubular structure obtained by a novel bioink. The developed bioink is composed of a porcine decellularized ECM obtained thus combining the main advantages of 3D bioprinting and the decellularization process.

Developing dECM gels could broaden the application of decellularized tissue, as gels can be used for cell encapsulation, bioactive molecules, and drugs delivery or injectable systems. Furthermore, dECM gels could be either an alternative to or incorporated into natural-based systems to provide a natural environment with bioactive molecules, to promote cellular adhesion, growth, and differentiation (Freytes et al., 2008). With the proposed bioink, we successfully printed tubular structures capable of maintaining the desired structure and avoiding the mixing of the bioinks with different colors, despite the increase in the number of layers. The parameter optimization allowed the fabrication of structures characterized by a height of 20 layers corresponding to 9 mm. Among the optimized parameters, the distance (n) between the layers of the construct is of paramount importance. It is essential to establish the exact value of n in which the layers do not overlap during printing to ensure stability and the achievement of the predetermined dimensions. The dimensions in mm of the internal (D_i_) and external (D_e_) diameter of each layer are reported in Table 2, considering different values of n. Furthermore, the extrusion height for each geometry was adapted to the dimensions of the nozzle 22 G, thus fixed at 0.41 mm. Moreover, the printing procedure was characterized by an extrusion pressure of 100 kPa and a velocity of 20 mm/s for both the printing heads.

**TABLE 2:** Dimensions of the internal (Di) and external (De) diameter of each layer, as a function of the parameter n, the distance between the layers of the construct.

Aorta	*n* = 0.5 mm	*n* = 0.7 mm	*n* = 1 mm
Intima layer (mm)	D_*i*_ = 20.00; D_*e*_ = 20.82	D_*i*_ = 20.00; D_*e*_ = 20.82	D_*i*_ = 20.00; D_*e*_ = 20.82
Media layer 1 (mm)	D_*i*_ = 21.82; D_*e*_ = 22.64	D_*i*_ = 22.22; D_*e*_ = 23.04	D_*i*_ = 22.82; D_*e*_ = 23.64
Media layer 2 (mm)	D_*i*_ = 23.64; D_*e*_ = 24.46	D_*i*_ = 24.44; D_*e*_ = 25.26	D_*i*_ = 25.64; D_*e*_ = 26.46
Adventitia layer (mm)	D_*i*_ = 25.46; D_*e*_ = 26.28	D_*i*_ = 26.66; D_*e*_ = 27.48	D_*i*_ = 28.46; D_*e*_ = 29.28

Comparing the dimensions of the design and the printed structures, with n set at 1 mm, the measurements resulted very close. The difference consists of 20% in internal diameter, 14% in height, and 8% in thickness (Table 1). The printed structure exhibited good structural stability and elasticity even when it reached a height of about 10 mm, making it possible to replicate the three different layers. The construct endured multiple manual deformation cycles.

The possibility to have confined layers is a key point to fabricate cellularized multi-layer structures. In particular, the endothelial cells were encapsulated in the developed bioink to print a cellularized tubular construct. In fact, the analysis of cell viability in the 3D bioprinted constructs, performed using a Live/Dead kit, demonstrated that this novel bioink is biocompatible and cytocompatible. Moreover, we can also appreciate the cell infiltration and growth through the images acquired after 15 and 20 days of culture. The proposed bioink is now ready to host other two different cell lines and study their behavior according to the architecture: in particular, endothelial cells to replicate the intima layer and smooth muscle cells for the media layer. Producing the re-endothelialization of the lumen surface is key to promote vascular repair and, in addition, smooth muscle cells are of fundamental importance due to their effects on the mechanical property and ECM deposition (Yuan et al., 2020).

The final goal is to obtain an engineered large blood vessel, with a structure similar to the native architecture in terms of cellular and extracellular components, with adequate mechanical properties and compatible with suturability.

In this research, first, we optimized a suitable decellularization strategy in order to produce a dECM. Then, we developed a novel cellularized bioink able to print tubular functional constructs with high resolution. This bioink could be employed to mimic various tissues such as blood vessels and the trachea.

## Data Availability

The datasets generated during and/or analyzed during the present study are available upon reasonable request to the corresponding author.
